# The Cunningham Panel is an unreliable biological measure

**DOI:** 10.1038/s41398-019-0413-x

**Published:** 2019-01-31

**Authors:** Susanne Bejerot, Eva Hesselmark

**Affiliations:** 1 Department of Psychiatry, School of Medical Sciences, Örebro University, Örebro, 701 82 Sweden; 2 University Health Care Research Center, Faculty of Medicine and Health, Örebro University, Örebro, Sweden; 3 Department of Clinical Neuroscience, Center for Psychiatry Research, Karolinska Institutet, Solna, Sweden; 4 Stockholm Health Care Services, Stockholm County Council, Stockholm, Sweden

## The Cunningham Panel is an unreliable biological measure

We read Connery et al. open-label case series entitled *Intravenous immunoglobulin for the treatment of autoimmune encephalopathy in children with autism*^1^ with great interest. It is undoubtedly important to communicate that children with autism may improve from IVIG treatment; this should be further investigated. In the study, the Cunningham Panel^2^ was used as a biological marker to predict improvement in the children. This immune panel comprises of five analytes: calcium calmodulin dependent kinase II activation (CamKII activation) and antibodies to dopamine receptors D1 and D2, β-tubulin, and lysoganglioside-GM1. CaMKII activation is regarded as positive if the activity exceeds 129, which was—in addition to one or more positive autoantibody titer—the prerequisite for IVIG treatment. The antidopamine D2L receptor antibody, the anti-tubulin antibody and the ratio of the antidopamine D2L to D1 receptor antibodies were chosen as predictors for treatment response. Improvement was based on the behavioral measures Aberrant Behavior Checklist (ABC) and/or Social Responsiveness Scale (SRS), in addition to parents’ reports.

According to the outcome measures, 52–65% of the children were responders to IVIG. Unfortunately, the biomarkers and the two behavioral measures ABC and SRS were only acquired in 16 of the 31 children before and after IVIG treatment. However, based on this limited information, the authors, nevertheless, conclude that the Cunningham Panel predicts response to IVIG treatment with good accuracy. Yet, it is unknown if children with CaMKII activity below 130 would similarly improve from IVIG treatment, since the 17 patients reported to have such test results were not treated with IVIG. It could also well be that children with autism and normal levels of autoantibodies against dopamine receptors D1 and D2, β-tubulin, and lysoganglioside-GM1 may improve from IVIG treatment; this was not investigated in the study. Therefore, it remains unknown if any of the analytes in the Cunningham Panel can predict treatment response.

We have previously questioned the diagnostic value of the Cunningham Panel^3,4^. In a Swedish study consisting of 53 patients (40 children and adolescents and 13 adults) with suspicion of having pediatric acute neuropsychiatric syndrome (PANS) and/or pediatric autoimmune neuropsychiatric disorders associated with streptococcal infections (PANDAS), the property of the Cunningham Panel was investigated. To our knowledge, our study is the only independent study evaluating the Cunningham Panel hitherto published. Twenty-four of the patients met diagnostic criteria for PANS and/or PANDAS, whereas 29 did not. We also tested 27 healthy controls with the Cunningham Panel. Ten out of 21 (48%) healthy controls had elevated CaMKII activity as compared to 35 out of 53 (66%) of the patients. Moreover, at least one positive autoantibody titer was found in 17 of the healthy controls. In conclusion we found a so-called positive value on the Cunningham Panel in 86% of the healthy controls, as compared to 92% in the patients assessed for PANS and PANDAS.

To summarize, Connery et al. do not present any objective data in the causal pathway from symptoms to outcomes. Seveteen children with autism in the study were not treated with IVIG on the basis that their CaMKII was not elevated. Consequently, the effect of IVIG in children with autism and subthreshold levels of CaMKII activity is not tested. The reported improvements in the children with elevated CaMKII activity may stem from placebo response or the natural course, not necessarily from IVIG. Considering that the Cunningham Panel to a great extent is positive among healthy people, it seems premature to suggest that it can predict treatment response based on a small selection of children with autism in an open-label, uncontrolled study. At this point of time we cannot draw any quantitative conclusions about biomarkers from open label treatment studies of disorders like ASD.
